# Exercise Hypertension in Athletes

**DOI:** 10.3390/jcm11164870

**Published:** 2022-08-19

**Authors:** Karsten Keller, Katharina Hartung, Luis del Castillo Carillo, Julia Treiber, Florian Stock, Chantal Schröder, Florian Hugenschmidt, Birgit Friedmann-Bette

**Affiliations:** 1 Medical Clinic VII, Department of Sports Medicine, University Hospital Heidelberg, 69120 Heidelberg, Germany; 2 Department of Cardiology, University Medical Center of the Johannes Gutenberg-University Mainz, 55131 Mainz, Germany; 3 Center for Thrombosis and Hemostasis (CTH), University Medical Center of the Johannes Gutenberg-University Mainz, 55131 Mainz, Germany

**Keywords:** arterial hypertension, exercise hypertension, blood pressure, exercise testing

## Abstract

**Background:** An exaggerated blood pressure response (EBPR) during exercise testing is not well defined, and several blood pressure thresholds are used in different studies and recommended in different guidelines. **Methods:** Competitive athletes of any age without known arterial hypertension who presented for preparticipation screening were included in the present study and categorized for EBPR according to American Heart Association (AHA), European Society of Cardiology (ESC), and American College of Sports Medicine (ACSM) guidelines as well as the systolic blood pressure/MET slope method. **Results:** Overall, 1137 athletes (mean age 21 years; 34.7% females) without known arterial hypertension were included April 2020–October 2021. Among them, 19.6%, 15.0%, and 6.8% were diagnosed EBPR according to ESC, AHA, and ACSM guidelines, respectively. Left ventricular hypertrophy (LVH) was detected in 20.5% of the athletes and was approximately two-fold more frequent in athletes with EBPR than in those without. While EBPR according to AHA (OR 2.35 [95%CI 1.66–3.33], *p* < 0.001) and ACSM guidelines (OR 1.81 [95%CI 1.05–3.09], *p* = 0.031) was independently (of age and sex) associated with LVH, EBPR defined according to ESC guidelines (OR 1.49 [95%CI 1.00–2.23], *p* = 0.051) was not. In adult athletes, only AHA guidelines (OR 1.96 [95%CI 1.32–2.90], *p* = 0.001) and systolic blood pressure/MET slope method (OR 1.73 [95%CI 1.08–2.78], *p* = 0.023) were independently predictive for LVH. **Conclusions:** Diverging guidelines exist for the screening regarding EBPR. In competitive athletes, the prevalence of EBPR was highest when applying the ESC (19.6%) and lowest using the ACSM guidelines (6.8%). An association of EBPR with LVH in adult athletes, independently of age and sex, was only found when the AHA guideline or the systolic blood pressure/MET slope method was applied.

## 1. Introduction

Arterial hypertension is the most important and most common cardiovascular risk factor (CVRF) for morbidity and mortality worldwide [1,2,3,4]. The prevalence of arterial hypertension is high [5], affecting approximately 78 million adults in the United States of America [6]. While the prevalence of arterial hypertension increases substantially with age [7,8,9,10], its prevalence in athletes is low, at approximately 3% [11].

Diagnosis of arterial hypertension by resting blood pressure is well defined. In Europe, a systolic blood pressure (BP) value of ≥140 mmHg and a diastolic BP value of ≥90 mmHg are the defined thresholds of arterial hypertension [12,13,14,15]. In contrast, an exaggerated blood pressure response (EBPR) during treadmill and bicycle exercise testing is not well defined and poorly recognized, and several blood pressure thresholds were used in the different studies and are recommended in different guidelines [9,14,16,17,18,19,20,21,22]. While the American Heart Association (AHA) guideline [23] (EBPR threshold: systolic peak BP >210 mmHg in men, >190 mmHg in women, and/or >90 mmHg diastolic peak BP in both sexes) and the European Society of Cardiology (ESC) guideline [22,24] (EBPR threshold: systolic peak BP >220 mmHg in men, >200 mmHg in women, and/or >85 mmHg in men and 80 mmHg in women for diastolic peak BP) used sex-specific EBPR thresholds, the American College of Sports Medicine (ACSM) guideline [20,21] (EBPR threshold: systolic peak BP >225 mmHg and/or >90 mmHg for diastolic peak BP in both sexes) recommends the same systolic and diastolic thresholds values for both sexes.

However, for arterial-hypertension-naïve individuals with EBPR during the exercise testing, it was shown that these individuals are at increased risk of developing both arterial hypertension as well as cardiovascular events in the future, underlining the importance of this phenomenon [1,4,17,25,26,27,28,29,30,31,32,33,34,35,36,37].

In the context of arterial hypertension, it is well known that an increase in left ventricular mass and left ventricular hypertrophy (LVH) are associated with cardiovascular disease (CVD) as well as an elevated number of cardiovascular events and mortality [37,38]. Despite the development of the heart in highly trained athletes, a septal thickness of ≥13 mm was observed in only a very small number of athletes and should be considered as LVH in athletes [22,39,40,41].

Thus, the objectives of the present study were to evaluate (I) how prevalent EBPR is in athletes and (II) which definition of an EBPR during exercise testing was best associated with LVH in athletes without known arterial hypertension.

## 2. Materials and Methods

We performed a retrospective analysis of athletes of any age without known arterial hypertension who presented at the Department of Sports Medicine (Medical Clinic VII) of the University Hospital Heidelberg (Germany) for their preparticipation screening examination between April 2020 and October 2021.

### 2.1. Enrolled Subjects

Athletes were eligible for this study if they performed regular training for competition, were able to perform an exercise test at our department, had no contraindications for exercise testing, and had no known diagnosis of arterial hypertension. Exclusion criteria were a known diagnosis of arterial hypertension and contraindications regarding performing exercise testing [22,23].

### 2.2. Ethical Aspects

The requirement for informed consent was waived as we used only anonymized retrospective data routinely collected during the health screening process. Studies in Germany involving a retrospective analysis of diagnostic standard data of anonymized patients do not require an ethics statement.

### 2.3. Definitions

Arterial hypertension at rest was defined according to the ESC guidelines [42]. In all athletes, a transthoracic echocardiography was performed. Investigated echocardiographic parameters were defined according to current guidelines [22,43].

LVH was defined as (I) septal or posterior left ventricular (LV) wall diameter ≥13 mm [22,40] or (II) LV mass >162 g in female or >224 g in male individuals [43]. LV mass was computed according the established 2D echocardiography area-length method: LV mass = 0.80 × (1.04 × [(septal LV wall thickness + LV end-diastolic diameter + posterior LV wall thickness)^3^ − (LV end-diastolic diameter)^3^]) + 0.6 g [43]. LVH was considered to be present if one or both of the definitions applied.

EBPR was defined on the basis of the peak BP values during exercise testing according to three different guidelines and the systolic BP/MET slope method:

- American Heart Association (AHA) guidelines [23]: systolic peak BP >210 mmHg in men, >190 mmHg in women, and/or >90 mmHg diastolic peak BP in both sexes.
- European Society of Cardiology (ESC) guidelines [22,24]: systolic peak BP >220 mmHg in men, >200 mmHg in women, and/or >85 mmHg in men and 80 mmHg in women for diastolic peak BP.
- The American College of Sports Medicine (ACSM) guidelines [20,21]: systolic peak BP >225 mmHg and/or >90 mmHg, for diastolic peak BP in both sexes.
- The systolic BP/MET slope method [44,45,46,47]: The Δ regarding systolic BP was calculated as maximum systolic BP during exercise—systolic BP at rest and was indexed by the increase in MET from rest (Δ regarding MET was calculated as peak MET-1) to obtain the systolic BP/MET slope [46]. In accordance with previous studies, a cutoff value > 6.2 mmHg/MET was used to define an EBPR [44,46]. The MET value was calculated based on the athletes’ VO_2_ maximum values during exercise testing as recommended by the ACSM guideline (MET = VO_2max_/3.5 mL·kg^−1^·min^−1^) [48].
- Exercise testing was performed according to current guidelines with electrocardiogram (ECG) and BP measurements at the end of every load level. The exercise test was stopped if the athlete was at their maximum capacity or stopping criteria according to current guidelines [22,23].

Obesity was defined as body mass index (BMI) ≥30 kg/m^2^ according to the World Health Organization.

### 2.4. Statistics

Athletes categorized as athletes with EBPR according to the three aforementioned guidelines and the systolic BP/MET slope method were compared to those athletes not categorized as EBPR (normal BP response during the exercise test) with the help of the Wilcoxon–Mann–Whitney U test for continuous variables and Fisher’s exact or chi^2^ test for categorical variables, as appropriate. Data of continuous variables were presented as median and interquartile range and categorical variables as absolute numbers with related percentages.

We performed univariate and multivariate logistic regression models to investigate the association between EBPR (defined according to the three guidelines) as well as BP values at rest and maximum values during exercise on the one hand and LVH on the other hand. Multivariate regression models were adjusted for age and sex in order to prove the independence of the statistical results of athletes’ age and sex. Results of the logistic regressions are presented as odds ratio (OR) and 95% Confidence interval (CI).

All statistical analyses were carried out with the use of SPSS software (IBM Corp. Released 2017. IBM SPSS Statistics for Windows, Version 25.0. Armonk, NY, USA). Only the *p* values < 0.05 (two-sided) were considered to be statistically significant. No adjustment for multiple testing was applied to the present analysis.

## 3. Results

### 3.1. Athletes’ Characteristics

**Figure 1:**
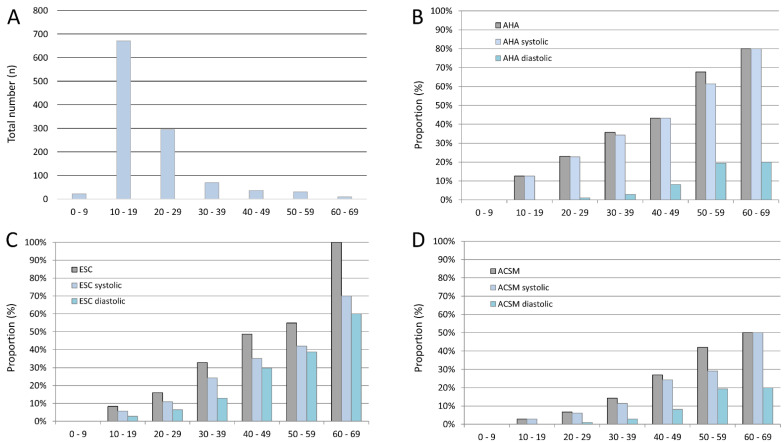
Included numbers of athletes and proportion of blood pressure deviations stratified for age by decade. Panel (**A**) Total numbers of included athletes stratified for age by decade. Panel (**B**) Proportion of athletes with exaggerated blood pressure response according to American Heart Association (AHA) guideline stratified for age by decade. Panel (**C**) Proportion of athletes with exaggerated blood pressure response according to European Society of Cardiology (ESC) guideline stratified for age by decade. Panel (**D**) Proportion of athletes with exaggerated blood pressure response according to American College of Sports Medicine (ACSM) guideline stratified for age by decade.

Overall, 1137 athletes (mean age 21 years; median 18 years (IQR 15/25); 395 (34.7%) females) without known arterial hypertension were included in the present study between April 2020 and October 2021. Most included athletes were in the second or third decade of life (Figure 1A). Among them, CVRF were rare, with nicotine abuse reported in 34 (3.0%) and obesity detected in 14 (1.2%) athletes. LVH was diagnosed in 233 athletes (regardless of athletes’ sex: 20.5%; 87 female athletes (22.0%); 146 male athletes (19.7%)). Median past training period was 8 (IQR 5/12) years.

### 3.2. Prevalence of Exaggerated Blood Pressure Response (EBPR) during Exercise Testing

Overall, 223 athletes (regardless of athletes’ sex: 19.6%; 74 female athletes (18.7%); 149 male athletes (20.1%)) had a diagnosis of EBPR according to AHA guidelines (Table 1), 171 (regardless of athletes’ sex: 15.0%; 66 female athletes (16.7%); 105 male athletes (14.2%)) according to ESC guidelines (Table 2), and 77 (regardless of athletes’ sex: 6.8%; 11 female athletes (2.8%); 66 male athletes (8.9%)) according to ACSM guidelines (Table 3).

### 3.3. Comparison of Athletes with and without Exaggerated Blood Pressure Response (EBPR) during Exercise Testing

While the proportions of female athletes with and without EBPR according to ESC and AHA guidelines were widely balanced, comprising approximately 1/3 of the athletes with EBPR, the proportion of male athletes with EBPR according to ACSM was distinctly higher, with 85.7% of all individuals with EBPR (Table 3). CVRF nicotine abuse and obesity were both more prevalent in athletes with EBPR regardless of which definition of EBPR was chosen (Table 1, Table 2 and Table 3). The criteria regarding full effort during the exercise test did not differ between athletes with and without EBPR (Table 1, Table 2 and Table 3).

The proportion of athletes with EBPR increased with inclining age regardless of the chosen definition. Notably, EBPR was more often diagnosed due to maximum systolic in comparison to maximum diastolic blood pressure values during exercise (Figure 1B–D).

### 3.4. Prevalence of Left Ventricular Hypertrophy (LVH) in Athletes

LVH was approximately two-fold more frequent in athletes with EBPR than in those without (risk ratios (RR) 2.2, 1.8, and 2.0 when using the definitions of AHA guidelines, ESC guidelines, and ACSM guidelines, respectively).

Interestingly, aortic valve regurgitation and mitral valve regurgitation were both more prevalent in athletes with EBPR (Table 1, Table 2 and Table 3).

### 3.5. Association of Exaggerated Blood Pressure Response (EBPR) during Exercise Testing and Left Ventricular Hypertrophy (LVH) in Athletes 

**Figure 2:**
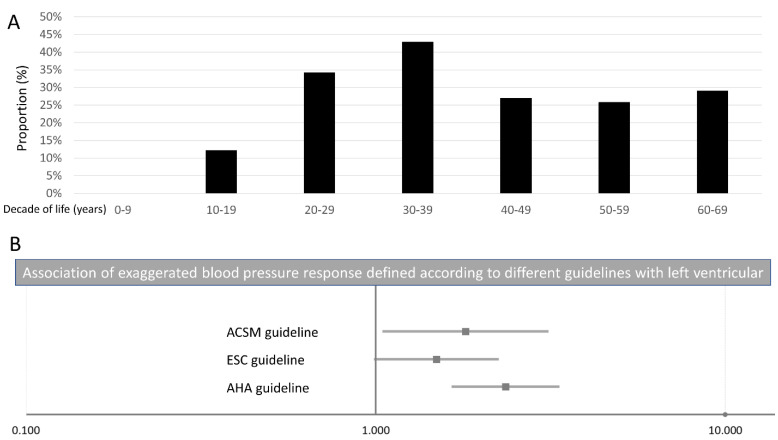
Exaggerated blood pressure response and left ventricular hypertrophy. Panel (**A**) Proportion of left ventricular hypertrophy stratified for age by decades. Panel (**B**) Association of exaggerated blood pressure response according to AHA, ESC, and ACSM guidelines with left ventricular hypertrophy.

In addition, we computed logistic regression models in order to analyse associations between EBPR defined according to the different guidelines on the one hand and LVH on the other hand. While EBPR according to the definition of the AHA guidelines (OR 2.35 (95%CI 1.66–3.33), *p* < 0.001) and the ACSM guidelines (OR 1.81 (95%CI 1.05–3.09), *p* = 0.031) were independently (of age and sex) associated with LVH, EBPR defined according to the ESC guidelines (OR 1.49 (95%CI 1.00–2.23), *p* = 0.051) was not independently associated with LVH (Figure 2B, Table 4).

In addition, LVH was associated with systolic BP at rest and maximum systolic BP during exercise, but not with diastolic BP values (Table 4).

### 3.6. Prevalence of Exaggerated Blood Pressure Response (EBPR) during Exercise Testing and Left Ventricular Hypertrophy (LVH) in Adult Athletes 

When focusing on the adult athletes only, 598 athletes (33.1% females; median age 23.0 (19.0–29.0) years) aged 18 years or older remained in the analysis. Among these, 180 (30.1%) had an LVH.

According to the guideline definitions, 170 (regardless of athletes’ sex: 28.4%; 54 female athletes (27.3%); 116 male athletes (29.0%)) athletes were classified as EBPR according to AHA guidelines, 137 (regardless of athletes’ sex: 22.9%; 54 female athletes (27.3%); 83 male athletes (20.8%)) according to ESC guidelines, and 65 (regardless of athletes’ sex: 10.9%; 11 female athletes (5.6%); 54 male athletes (13.5%)) according to ACSM guidelines.

### 3.7. Association of Exaggerated Blood Pressure Response (EBPR) during Exercise Testing and Left Ventricular Hypertrophy (LVH) in Adult Athletes 

In adult athletes, only the definition of EBPR according to AHA guidelines was independently predictive for LVH (univariate: OR 1.88 (95%CI 1.29–2.74), *p* = 0.001; multivariate: OR 1.96 (95% CI 1.32–2.90), *p* = 0.001). EBPR according to the ESC (univariate: OR 1.40 (95% CI 0.94–2.10), *p* = 0.100; multivariate: OR 1.44 (95%CI 0.93–2.22), *p* = 0.104) as well as ACSM guidelines (univariate: OR 1.64 (95% CI 0.97–2.79), *p* = 0.067; multivariate: OR 1.73 (95% CI 0.98–3.07), *p* = 0.060) were not associated with LVH independently of age and sex.

### 3.8. Prevalence of Exaggerated Blood Pressure Response (EBPR) during Exercise Testing Identified by Systolic BP/MET Slope Method with a Cutoff Value > 6.2 mmHg/MET 

When using the systolic BP/MET slope method with a cutoff value > 6.2 mmHg/MET to define an EBPR in those 639 athletes, who underwent spiroergometric testing, we detected 386 athletes (60.4%) with normal BP response and 253 athletes with EBPR (regardless of athletes’ sex: 39.6%; 80 female athletes (36.5%); 173 male athletes (41.2%)) (Table 5). LVH was more prevalent in athletes with than without EBPR (29.6% vs. 16.6%, *p* < 0.001).

### 3.9. Association of Exaggerated Blood Pressure Response (EBPR) during Exercise Testing Identified by Systolic BP/MET Slope Method with a Cutoff Value > 6.2 mmHg/MET and Left Ventricular Hypertrophy (LVH) in Athletes 

Systolic BP/MET slope > 6.2 mmHg/MET was associated with LVH in the univariate regression analysis (OR 2.12 (95% CI 1.45–3.10), *p* < 0.001), but this association remained not significant after adjustment for age and sex (OR 2.26 (95% CI 0.40–12.66), *p* = 0.355). Sex-specific analyses revealed a significant association of systolic BP/MET slope > 6.2 mmHg/MET with LVH in male (OR 2.348 (95%CI 1.472–3.746), *p* < 0.001) in contrast to female athletes (OR 1.706 (95%CI 0.878–3.315), *p* = 0.115).

In contrast, in the 398 adult athletes with spiroergometric evaluation, systolic BP/MET slope > 6.2 mmHg/MET was associated with LVH in both, the univariate (OR 1.67 (95% CI 1.07–2.60), *p* = 0.023) as well as multivariate logistic regression analysis adjusted for age and sex (OR 1.73 (95% CI 1.08–2.78), *p* = 0.023). However, sex-specific analyses also revealed sex-specific differences in adult athletes. While systolic BP/MET slope > 6.2 mmHg/MET was associated with LVH in male adult athletes (OR 1.848 (95% CI 1.079–3.166), *p* = 0.025), in females, no association was seen (OR 1.325 (95% CI 0.603–2.913), *p* = 0.484).

## 4. Discussion

Arterial hypertension is accompanied by substantially increased cardiovascular morbidity and mortality [2,4,7,9,17,49,50,51].

Among individuals who were not categorized as patients with arterial hypertension [12,13,14,15] a number of individuals revealed EBPR during exercise testing. The consequences of this phenomenon are not well elucidated, and study results are inconsistent. In previous investigations, a large number of different definitions of EBPR were used, hampering a clear interpretation of study results [1,4,17,25,26,27,28,29,30,31,32,33,34,35,36,37]. However, several studies have shown that individuals without known arterial hypertension who present with EBPR during the exercise testing are at increased risk to develop arterial hypertension in the future and might also be prone to develop cardiovascular events [1,4,17,25,26,27,28,29,30,31,32,33,34,35,36,37]. Three guideline definitions are currently available and valid: the AHA [23], the ESC [22,24], and the ACSM guidelines [20,21]. In this context, it is widely unclear from which study sample these definitions were derived and whether these definitions were able to predict cardiovascular morbidity, e.g., LVH, in athletes.

Thus, the objectives of our present study were to evaluate the prevalence of EBPR in athletes and which definition regarding EBPR during exercise testing was best/strongest associated with LVH in athletes without known arterial hypertension.

The main results of the study can be summarized as follows:

(I) EBPR was diagnosed between 6.8% and 19.6% of all athletes in our study according to the different guideline recommendations. Prevalence was highest when categorized according to the ESC guidelines (19.6%) and lowest according to the ACSM guidelines (6.8%).
(II) CVRF, such as nicotine abuse and obesity, were more prevalent in athletes with EBPR.
(III) The proportion of athletes with EBPR increased with inclining age regardless of the chosen definition.
(IV) EBPR was more often diagnosed due to maximum systolic in comparison to maximum diastolic BP values during exercise.
(V) Only the EBPR definition of the AHA guideline was able to predict LVH independently of age and sex in both the overall sample as well as in adult athletes as the only guideline recommended threshold.
(VI) In addition, the recently implemented systolic BP/MET slope method with a cutoff value > 6.2 mmHg/MET to define an EBPR, was able to predict LVH in adult athletes independently of age and sex.

Our study results reveal a large variation regarding the prevalence of EBPR according to the different guideline definitions in athletes without known arterial hypertension (variation of 12.8% according to different guideline recommendations). The prevalence was highest when categorized according to the ESC guidelines [22,24] (19.6%) and lowest when classified according to the ACSM guidelines [20,42] (6.8%). In contrast to the study of Caselli at al. [24], who reported that only a rate of 7.5% of the 1876 investigated athletes had an EBPR defined according to the ESC guidelines, we identified a frequency of 19.6% in the athletes presenting with EBPR according the ESC guidelines’ definition. However, the differences between our results and the aforementioned study might be based on differences regarding the performance level of the examined athletes and athletes’ ages in both studies.

As expected, CVRF, such as nicotine abuse and obesity, were in our study more prevalent in those athletes with EBPR. This finding is in line with the literature, reporting a close relation between obesity and elevated blood pressure [52,53]. Arterial hypertension is frequently observed in individuals who are obese [53]. In addition, smoking was strongly associated with arterial hypertension in several studies [54,55].

The proportion of athletes with EBPR increased significantly with inclining age regardless of the chosen definition. In this context, studies underlined a physiological increase in BP with age [4,56,57,58]. While at birth, the systolic and diastolic BP values are on average at levels of 70 mmHg and 50 mmHg, respectively [4,56,58], BP values rise progressively throughout childhood and adolescence [4,56,57,58]. As aforementioned, BP is substantially determined by body weight, and it is of key interest that BP in childhood has a strong impact on adult BP levels [4,57,58]. Individuals aged ≥70 years reach an average systolic BP of approximately 140 mmHg. Diastolic BP tends also to rise with the aging process but the intense of this increase is less steep and after the 50th life year, diastolic mean BP either inclines only slightly or even declines [4,56]. These changes in BP reflect normal age-dependent development, while BP deviations due to arterial hypertension could be detected in every period of life [4,56]. The association between a growing burden of arterial hypertension with increasing age is well known and described [4,6,56,59]. While in Germany, 10–35% of the citizens aged between 30 and 60 were diagnosed with arterial hypertension, the frequency increases to higher than 65% in people aged 60 years and older [8]. In light of the quoted literature, an age-dependent increase regarding the proportion of athletes with EBPR might be expected but could also be interpreted as an increasing number of athletes who might have undiagnosed or masked arterial hypertension.

In stress situations, the BP rises from resting to stress level depending on the exercise intensity and the affecting stressor [4,17,19,60]. The BP responses to exercise are a result of cardiac output and peripheral vascular resistance [61]. Cardiac output is elevated to provide oxygenated blood and nutrition for the active regions of the body according to increased demand [62]. During physical activity, BP values increase, whereby the rise in systolic BP values becomes more pronounced compared to diastolic BP. BP values generally increase to an exercise dependent and predetermined individual limit [1,4,17,61]. Normal systolic BP response in progressive exercise testing on a bicycle stress test comprise a systolic BP increase of approximately 7 to 10 mmHg per 25 watt workload incline [19]. Expected maximal BP values in bicycle testing are approximately 200/100 mmHg in healthy untrained adults in the general population and approximately 215/105 mmHg in those individuals who are older than 50 years [16]. Notably, only systolic BP values, not diastolic values, could be reliably measured with the standardly used non-invasive methods [1].

Thus, in our present study, it is of outstanding importance that EBPR was more often diagnosed due to maximum systolic in comparison to maximum diastolic BP values during exercise, although all of the guideline recommendations defined a diastolic threshold regarding EBPR [20,21,22,23,24].

Although three different guideline recommendations for the definition of EBPR are available, only the EBPR definition of the AHA guidelines [23] was able to predict LVH independently of age and sex in both the overall sample as well as in adult athletes only in our study. Nevertheless, despite this result, we do not think that the definition of EBPR as systolic BP > 210 mmHg in men, > 190 mmHg in women, and/or > 90 mmHg diastolic peak BP in both sexes [23] is well suited to identify individuals at risk and deduce further consequences as a singular diagnostic tool in athletes. From the experiences of daily routine in sports medicine, the defined systolic BP values regarding EBPR are too low for exercise testing in male and female athletes. In accordance with these experiences of daily practice, it has been reported in the literature that very fit and powerful athletes reach physiologically higher BP values during competition as well as exercise testing [4,16,19,63]. Although, systolic BP values ≥ 250 mmHg and diastolic BP values ≥ 120 mmHg were defined as stopping criteria for bicycle ergometry exercise testing [16,63,64]—especially in young athletes, who exceed these thresholds within their normal sports practice—a stop of the exercise testing even at this higher and rigid recommended thresholds (250/120 mmHg) seems limited in its usefulness and the decision to stop should be made individually [16,19,63].

In order to encounter these only-in-part useful definitions of EBPR for athletes, a workload-indexed EBPR definition was introduced by different authors with promising results [44,45,46,47]. Our study confirmed these results—that an EBPR defined according to the systolic BP/MET slope method with a cutoff value > 6.2 mmHg/MET was able to predict LVH in adult athletes independently of age and sex. A threshold of 6.2 mmHg/MET was chosen since a systolic BP/MET slope >6.2 mmHg/MET was in the study of Hedman et al. associated with a 27% higher risk for mortality during a 20-year observational period in males compared to those with <4.3 mmHg/MET [44,46]. However, we detected sex-specific differences regarding this associations between EBPR defined according to the systolic BP/MET slope method with a cutoff value >6.2 mmHg/MET and LVH with significant associations in males and missing associations in females. In accordance, several studies revealed sex-specific differences regarding blood pressure response in males and females [65,66,67]. In studies, men had significantly higher systolic BP values at 50%, 75%, and 100% of maximum exercise efforts [67].

Nevertheless, although these recommended EBPR thresholds—defined by the three guidelines—seem only in part to be suitable for athletes (but more for the general untrained population), an identified EBPR and especially a prolonged and delayed decline in blood pressure after exercise testing could provide clues regarding a masked arterial hypertension or development of a manifest arterial hypertension in the future [4,63].

In athletes with EBPR and/or a prolonged and delayed decline in blood pressure after exercise testing, a 24 h blood pressure measurement could give important and valuable additional diagnostic information [15]. Where the threshold regarding EBPR in athletes from which further diagnostic procedures should be implemented is still controversial [16,19,63].

## 5. Conclusions

EBPR was diagnosed in between 6.8% and 19.6% of all athletes without known arterial hypertension. Prevalence was highest when athletes were categorized according to ESC guidelines (19.6%) and lowest when categorized according to ACSM guidelines (6.8%). The proportion of athletes with EBPR increased with inclining age regardless of the chosen definition. Only the EBPR definition of the AHA guidelines and the systolic blood pressure/MET slope method were associated with LVH independently of age and sex in adult athletes. However, the prognostic value of this association remains to be elucidated by sufficiently powered in-depth long-term studies. Such studies are also necessary to further evaluate the importance of the identification of EBPR in athletes and the significance of actual EBPR guidelines as diagnostic tools in young athletes.

## Figures and Tables

**Table 1:** Patient characteristics of the 1137 examined athletes without known arterial hypertension stratified for exaggerated blood pressure response according to AHA guideline.

Parameters	Normal Blood Pressure Response According to AHA Classification (*n* = 914; 80.4%)	Exaggerated Blood Pressure Response According to AHA Classification (*n* = 223; 19.6%)	*p*-Value
Age (in years)	17.0 (15.0/22.0)	22.0 (18.0/33.0)	<0.001
Female sex	321 (35.1%)	74 (33.2%)	0.586
Body height (cm)	174.0 (166.9/181.0)	179.0 (173.0/184.0)	<0.001
Body weight (kg)	67.0 (57.6/77.7)	75.8 (68.0/85.8)	<0.001
Body mass index (kg/m^2^)	22.0 (20.2/24.1)	23.4 (22.0/25.4)	<0.001
Body fat (%)	11.3 (8.5/16.4)	11.9 (9.0/16.3)	0.140
Leading athletes at a regional or national level	707 (77.4%)	146 (65.5%)	<0.001
Training years	8.0 (5.0/11.0)	11.0 (6.0/15.0)	<0.001
Cardiovascular risk factors			
Nicotine abuse	20 (2.2%)	14 (6.3%)	0.003
Obesity	8 (0.9%)	6 (2.7%)	0.039
Blood pressure values			
Systolic blood pressure (mmHg)	115.0 (110.0/120.0)	120.0 (115.0/130.0)	<0.001
Diastolic blood pressure (mmHg)	70.0 (60.0/75.0)	70.0 (70.0/80.0)	<0.001
Maximum systolic blood pressure during exercise (mmHg)	180.0 (160.0/190.0)	220.0 (210.0/230.0)	<0.001
Maximum diastolic blood pressure during exercise (mmHg)	70.0 (70.0/80.0)	80.0 (70.0/85.0)	<0.001
Exercise parameters			
VO_2_ maximum during exercise	45.5 (39.9/50.5)	44.0 (37.2/49.5)	0.031
Respiratory exchange ratio (RER)	1.15 (1.10/1.20)	1.15 (1.11/1.21)	0.864
Maximum lactate value	9.46 (7.79/11.2)	9.21 (7.61/11.24)	0.861
Echocardiographic parameters			
Left ventricular hypertrophy	151 (16.5%)	82 (36.8%)	<0.001
Left ventricular mass	158.8 (128.0/200.4)	194.2 (164.1/220.8)	<0.001
Aortic valve regurgitation	48 (5.3%)	26 (11.7%)	0.001
Mitral valve regurgitation	474 (51.9%)	153 (68.6%)	<0.001
Tricuspid valve regurgitation	115 (12.6%)	43 (19.3%)	0.027
Pulmonary valve regurgitation	91 (10.0%)	17 (7.6%)	0.311
Heart volume in total (mL)	760.5 (625.8/906.3)	910.3 (770.2/1004.5)	<0.001
Heart volume related to body weight (mL/kg)	11.4 (10.2/12.4)	11.7 (10.6/12.8)	0.003
Left ventricular ejection fraction (%)	65.0 (62.0/69.0)	66.0 (62.0/69.0)	0.140
Left ventricular end-diastolic diameter (cm)	49.0 (45.0/53.0)	51.0 (48.0/54.0)	<0.001
Left atrial area (cm^2^)	13.5 (11.1/15.4)	15.2 (12.9/17.6)	<0.001
Right atrial area (cm^2^)	13.2 (11.0/15.5)	15.1 (13.3/17.7)	<0.001
Tricuspid annular plane systolic excursion (TAPSE, cm)	2.46 (2.20/2.70)	2.6 (2.3/2.9)	<0.001
Systolic pulmonary artery pulmonary pressure (mmHg)	20.0 (17.0/23.0)	20.3 (17.0/23.6)	0.274
E/A quotient	2.7 (1.9/3.7)	2.6 (1.8/3.6)	0.215
E/E’ quotient	4.7 (4.0/5.7)	4.8 (4.0/5.7)	0.606

**Table 2:** Patient characteristics of the 1137 examined athletes without known arterial hypertension stratified for exaggerated blood pressure response according to ESC guideline.

Parameters	Normal Blood Pressure Response According to ESC Classification (*n* = 966; 85.0%)	Exaggerated Blood Pressure Response According to ESC Classification (*n* = 171; 15.0%)	*p*-Value
Age (in years)	17.0 (15.0/22.0)	26.0 (18.0/42.0)	<0.001
Female sex	329 (34.1%)	66 (38.6%)	0.251
Body height (cm)	175.0 (167.0/182.0)	179.0 (171.0/184.0)	<0.001
Body weight (kg)	68.2 (58.3/78.5)	75.8 (66.4/84.0)	<0.001
Body mass index (kg/m^2^)	22.1 (20.2/24.2)	23.7 (22.3/25.5)	<0.001
Body fat (%)	11.0 (8.5/16.0)	13.0 (9.5/17.2)	<0.001
Leading athletes at a regional or national level	754 (78.1%)	99 (57.9%)	<0.001
Training years	8.0 (5.0/11.0)	11.0 (7.0/16.0)	<0.001
Cardiovascular risk factors			
Nicotine abuse	19 (2.0%)	15 (8.8%)	<0.001
Obesity	8 (0.8%)	6 (3.5%)	0.011
Blood pressure values			
Systolic blood pressure (mmHg)	115.0 (110.0/120.0)	120.0 (110.0/130.0)	<0.001
Diastolic blood pressure (mmHg)	70.0 (60.0/75.0)	75.0 (70.0/80.0)	<0.001
Maximum systolic blood pressure during exercise (mmHg)	180.0 (160.0/195.0)	220.0 (210.0/230.0)	<0.001
Maximum diastolic blood pressure during exercise (mmHg)	70.0 (70.0/80.0)	85.0 (80.0/90.0)	<0.001
Exercise parameters			
VO_2_ maximum during exercise	45.6 (40.1/50.6)	42.0 (35.1/49.1)	<0.001
Respiratory exchange ratio (RER)	1.15 (1.10/1.20)	1.15 (1.11/1.21)	0.497
Maximum lactate value	9.42 (7.71/11.2)	9.28 (7.96/11.07)	0.933
Echocardiographic parameters			
Left ventricular hypertrophy	177 (18.3%)	56 (32.7%)	<0.001
Left ventricular mass	164.3 (132.6/200.8)	188.0 (153.2/219.7)	<0.001
Aortic valve regurgitation	50 (5.2%)	24 (14.0%)	<0.001
Mitral valve regurgitation	506 (52.4%)	121 (70.8%)	<0.001
Tricuspid valve regurgitation	123 (12.7%)	35 (20.5%)	0.022
Pulmonary valve regurgitation	94 (9.7%)	14 (8.2%)	0.526
Heart volume in total (mL)	774.4 (634.6/919.0)	883.0 (728.4/982.6)	<0.001
Heart volume related to body weight (mL/kg)	11.5 (10.3/12.5)	11.5 (10.3/12.5)	0.790
Left ventricular ejection fraction (%)	65.0 (62.0/68.0)	66.0 (63.0/69.0)	0.012
Left ventricular end-diastolic diameter (cm)	50.0 (46.0/53.0)	51.0 (47.0/54.0)	0.004
Left atrial area (cm^2^)	13.6 (11.3/15.6)	15.0 (12.6/15.6)	<0.001
Right atrial area (cm^2^)	13.4 (11.1/15.7)	15.0 (12.9/17.7)	<0.001
Tricuspid annular plane systolic excursion (TAPSE, cm)	2.50 (2.20/2.80)	2.6 (2.4/2.9)	<0.001
Systolic pulmonary artery pulmonary pressure (mmHg)	20.0 (17.0/23.0)	21.0 (18.0/24.1)	0.018
E/A quotient	2.7 (2.0/3.7)	2.2 (1.6/3.3)	<0.001
E/E’ quotient	4.7 (4.0/5.7)	4.9 (4.1/6.0)	0.167

**Table 3:** Patient characteristics of the 1137 examined athletes without known arterial hypertension stratified for exaggerated blood pressure response according to ACSM guideline.

Parameters	Normal Blood Pressure Response According to ACSM Classification (*n* = 1060; 93.2%)	Exaggerated Blood Pressure Response According to ACSM Classification (*n* = 77; 6.8%)	*p*-Value
Age (in years)	18.0 (15.0/23.0)	29.0 (19.5/48.5)	<0.001
Female sex	384 (36.2%)	11 (14.3%)	<0.001
Body height (cm)	175.0 (167.0/182.0)	181.0 (175.3/186.5)	<0.001
Body weight (kg)	68.4 (58.8/78.5)	80.3 (75.0/87.9)	<0.001
Body mass index (kg/m^2^)	22.2 (20.4/24.2)	24.4 (23.0/26.3)	<0.001
Body fat (%)	11.3 (8.6/16.7)	11.5 (9.2/14.0)	0.884
Leading athletes at a regional or national level	817 (77.1%)	36 (46.8%)	<0.001
Training years	8.0 (5.0/11.0)	13.0 (8.5/18.8)	<0.001
Cardiovascular risk factors			
Nicotine abuse	27 (2.5%)	7 (9.1%)	0.006
Obesity	10 (0.9%)	4 (5.2%)	0.012
Blood pressure values			
Systolic blood pressure (mmHg)	115.0 (110.0/120.0)	125.0 (120.0/135.0)	<0.001
Diastolic blood pressure (mmHg)	70.0 (60.0/75.0)	80.0 (70.0/80.0)	<0.001
Maximum systolic blood pressure during exercise (mmHg)	180.0 (160.0/200.0)	230.0 (230.0/240.0)	<0.001
Maximum diastolic blood pressure during exercise (mmHg)	75.0 (70.0/80.0)	80.0 (80.0/90.0)	<0.001
Exercise parameters			
VO_2_ maximum during exercise	45.4 (39.8/50.4)	43.2 (35.8/49.5)	0.040
Respiratory exchange ratio (RER)	1.15 (1.11/1.20)	1.15 (1.11/1.21)	0.515
Maximum lactate value	9.40 (7.75/11.21)	9.41 (7.85/11.16)	0.974
Echocardiographic parameters			
Left ventricular hypertrophy	203 (19.2%)	30 (39.0%)	<0.001
Left ventricular mass	164.3 (132.8/200.8)	207.1 (181.4/227.7)	<0.001
Aortic valve regurgitation	60 (5.7%)	14 (18.2%)	<0.001
Mitral valve regurgitation	571 (53.9%)	56 (72.7%)	0.001
Tricuspid valve regurgitation	141 (13.3%)	17 (22.1%)	0.090
Pulmonary valve regurgitation	101 (9.5%)	7 (9.1%)	1.000
Heart volume in total (mL)	774.6 (642.5/919.0)	965.4 (829.4/1047.0)	<0.001
Heart volume related to body weight (mL/kg)	11.5 (10.3/12.5)	11.7 (10.4/12.6)	0.350
Left ventricular ejection fraction (%)	65.0 (62.0/69.0)	66.0 (62.0/72.0)	0.037
Left ventricular end-diastolic diameter (cm)	49.0 (46.0/53.0)	52.0 (49.5/54.5)	<0.001
Left atrial area (cm^2^)	13.6 (11.4/15.7)	15.7 (14.4/18.2)	<0.001
Right atrial area (cm^2^)	13.5 (11.2/15.8)	16.5 (14.0/18.5)	<0.001
Tricuspid annular plane systolic excursion (TAPSE, cm)	2.50 (2.20/2.80)	2.6 (2.3/2.9)	0.001
Systolic pulmonary artery pulmonary pressure (mmHg)	20.0 (17.0/23.0)	22.0 (20.0/25.0)	<0.001
E/A quotient	2.7 (1.9/3.7)	2.1 (1.5/3.2)	<0.001
E/E’ quotient	4.7 (4.0/5.7)	5.1 (4.1/6.4)	0.080

**Table 4:** Association between of exaggerated blood pressure response, blood pressure values at rest, and maximum value during exercise on the one hand and left ventricular hypertrophy on the other hand (univariate and multivariate logistic regression model).

	Left Ventricular Hypertrophy			
	Univariate Regression Model		Multivariate Regression Model (Adjusted for Age and Sex)	
	OR (95% CI)	*p*-Value	OR (95% CI)	*p*-Value
AHA guideline classification of exaggerated blood pressure response	2.939 (2.127–4.060)	<0.001	2.351 (1.660–3.328)	<0.001
ESC guideline classification of exaggerated blood pressure response	2.171 (1.517–3.107)	<0.001	1.493 (0.998–2.232)	0.051
ACSM guideline classification of exaggerated blood pressure response	2.695 (1.663–4.367)	<0.001	1.805 (1.054–3.093)	0.031
Systolic blood pressure/MET slope (>6.2 mmHg/MET)	2.120 (1.449–3.101)	<0.001	2.257 (0.403–12.655)	0.355
Systolic blood pressure at rest (mmHg)	1.023 (1.010–1.036)	<0.001	1.016 (1.001–1.030)	0.033
Diastolic blood pressure at rest (mmHg)	1.025 (1.007–1.043)	0.005	1.011 (0.992–1.030)	0.253
Maximum systolic blood pressure during exercise (mmHg)	1.024 (1.018–1.030)	<0.001	1.026 (1.019–1.033)	<0.001
Maximum diastolic blood pressure during exercise (mmHg)	1.023 (1.007–1.040)	0.005	1.006 (0.989–1.024)	0.470

**Table 5:** Patient characteristics of the 639 examined athletes with spiroergometry and without known arterial hypertension stratified for exaggerated blood pressure response according to systolic blood pressure/MET slope.

Parameters	Normal Blood Pressure Response According to Systolic Blood Pressure/MET Slope (≤6.2 mmHg/MET) (*n* = 386; 60.4%)	Exaggerated Blood Pressure Response According to Systolic Blood Pressure/MET Slope (>6.2 mmHg/MET) (*n* = 253; 39.6%)	*p*-Value
Age (in years)	18.0 (15.0/22.0)	24.0 (18.0/36.5)	<0.001
Female sex	139 (36.0%)	80 (31.6%)	0.253
Body height (cm)	175.0 (168.0/182.0)	178.0 (170.0/184.0)	0.014
Body weight (kg)	66.8 (58.0/77.7)	76.0 (66.0/85.9)	<0.001
Body mass index (kg/m^2^)	21.7 (20.2/24.0)	23.8 (22.3/26.0)	<0.001
Body fat (%)	12.4 (8.2/16.6)	12.2 (9.2/17.1)	0.003
Leading athletes at a regional or national level	295 (76.4%)	135 (53.4%)	<0.001
Training years	7.0 (5.0/10.0)	10.0 (5.0/14.0)	<0.001
Cardiovascular risk factors			
Nicotine abuse	8 (2.1%)	18 (7.1%)	0.003
Obesity	1 (0.3%)	9 (3.6%)	0.001
Blood pressure values			
Systolic blood pressure (mmHg)	120.0 (110.0/125.0)	120.0 (110.0/125.0)	0.908
Diastolic blood pressure (mmHg)	70.0 (60.0/75.0)	70.0 (65.0/80.0)	0.003
Maximum systolic blood pressure during exercise (mmHg)	170.0 (155.0/180.0)	210.0 (190.0/220.0)	<0.001
Maximum diastolic blood pressure during exercise (mmHg)	70.0 (65.0/80.0)	80.0 (70.0/80.0)	<0.001
Exercise parameters			
VO_2_ maximum during exercise	47.5 (42.1/51.5)	41.9 (36.2/47.0)	<0.001
Respiratory exchange ratio (RER)	1.15 (1.10/1.19)	1.15 (1.11/1.21)	0.037
Maximum lactate value	9.36 (7.67/11.24)	9.51 (7.89/11.24)	0.533
Echocardiographic parameters			
Left ventricular hypertrophy	64 (16.6%)	75 (29.6%)	<0.001
Left ventricular mass	163.6 (132.3/199.3)	188.1 (153.4/220.6)	<0.001
Aortic valve regurgitation	20 (5.2%)	22 (8.7%)	0.080
Mitral valve regurgitation	203 (52.6%)	169 (66.8%)	<0.001
Tricuspid valve regurgitation	46 (12.0%)	51 (20.2%)	0.010
Pulmonary valve regurgitation	34 (8.8%)	25 (9.9%)	0.647
Heart volume in total (mL)	772.0 (639.0/908.5)	896.4 (732.9/1000.0)	<0.001
Heart volume related to body weight (mL/kg)	11.4 (10.2/12.4)	11.4 (10.2/12.3)	0.803
Left ventricular ejection fraction (%)	65.0 (62.0/69.0)	66.0 (63.0/69.0)	0.041
Left ventricular end-diastolic diameter (cm)	50.0 (46.0/53.0)	51.0 (47.0/54.0)	<0.001
Left atrial area (cm^2^)	13.5 (11.0/15.3)	14.9 (12.6/17.4)	<0.001
Right atrial area (cm^2^)	13.3 (11.1/15.5)	14.9 (12.8/17.9)	<0.001
Tricuspid annular plane systolic excursion (TAPSE, cm)	2.40 (2.20/2.70)	2.60 (2.40/2.90)	<0.001
Systolic pulmonary artery pulmonary pressure (mmHg)	20.0 (16.5/23.0)	21.5 (18.0/24.0)	0.002
E/A quotient	2.5 (1.9/3.4)	2.4 (1.6/3.6)	0.111
E/E’ quotient	4.7 (4.0/5.7)	4.9 (4.1/5.9)	0.193

## Data Availability

The data presented in this study are available upon request from the corresponding author.
